# A Case of Pulmonary Hypertensive Crisis Following Treatment of Retinopathy of Prematurity in an Extremely Preterm Infant

**DOI:** 10.1111/ped.70558

**Published:** 2026-10-13

**Authors:** Naofumi Hanyu, Shonosuke Nara, Toshihiro Nakayama, Gaku Yamanaka

**Affiliations:** ^1^ Department of Pediatrics and Adolescent Medicine Tokyo Medical University Tokyo Japan

**Keywords:** bronchopulmonary dysplasia, extremely preterm infant, pulmonary hypertension, retinopathy of prematurity, ventilation‐perfusion ratio

Bronchopulmonary dysplasia–associated pulmonary hypertension (BPD‐PH) is characterized by abnormal development of the alveoli and pulmonary microvasculature and is associated with increased mortality and poor neurodevelopmental outcomes. We report a case of an acute pulmonary hypertensive crisis following intravitreal ranibizumab injection in an extremely preterm infant with severe bronchopulmonary dysplasia. Risk factors for BPD‐PH include extreme prematurity, prolonged mechanical ventilation, intraventricular hemorrhage, systemic corticosteroid exposure, hyperoxia, and inflammation or sepsis [1]. Despite awareness of these risk factors, the prediction and prevention of BPD‐PH remain difficult.

The patient was the second of dichorionic diamniotic twins from a pregnancy achieved via frozen embryo transfer and was delivered by cesarean section at 24 weeks and 1 day because of severe fetal growth restriction. He was classified as small for gestational age, with a birth weight of 437 g (< 3rd percentile). Placental pathology revealed Stage II chorioamnionitis according to the Blanc classification [2]. The Apgar scores were 5 at 1 min and 7 at 5 min. Surfactant therapy and invasive mechanical ventilation were started for neonatal respiratory distress syndrome. Inhaled nitric oxide (iNO) was initiated on postnatal day 0 and administered for 3 days to treat persistent pulmonary hypertension of the newborn. Hemodynamically significant patent ductus arteriosus was surgically clipped at a postmenstrual age (PMA) of 26 weeks. At a PMA of 33 weeks, he was extubated and transitioned to noninvasive ventilatory support, including synchronized intermittent positive airway pressure and a high‐flow nasal cannula.

Echocardiography performed at a PMA of 34 weeks revealed no echocardiographic findings suggestive of pulmonary hypertension. At a PMA of 35 weeks (body weight, 1303 g), he was reintubated for bilateral intravitreal injection of ranibizumab (0.2 mg/eye) to treat retinopathy of prematurity (Zone II, Stage 2 with plus disease). He was managed by synchronized intermittent mandatory ventilation with pressure support as follows: peak inspiratory pressure, 18 cmH_2_O; positive end‐expiratory pressure, 5 cmH_2_O; respiratory rate, 40 breaths per minute; inspiratory time, 0.45 s; pressure support level, 5 cmH_2_O. Pulmonary hemorrhage did not occur during reintubation or the subsequent clinical course. Acute hypoxemia developed 12 h after the injection. PH crisis was diagnosed by echocardiography. The chest radiograph obtained immediately before the onset, along with the subsequent clinical course and management, is shown in Figure 1A–C. Respiratory severity score correlates with severe BPD, mortality, and clinical outcomes [3]. The management strategies included iNO and a pulmonary vasodilator, namely, prostacyclin (epoprostenol), a phosphodiesterase Type 5 inhibitor (sildenafil), and diuretic therapy (furosemide, spironolactone). He was placed on fully controlled mechanical ventilation with sedation, analgesia, and neuromuscular blockade. The PH stabilized after approximately 2 months.

**FIGURE 1:**
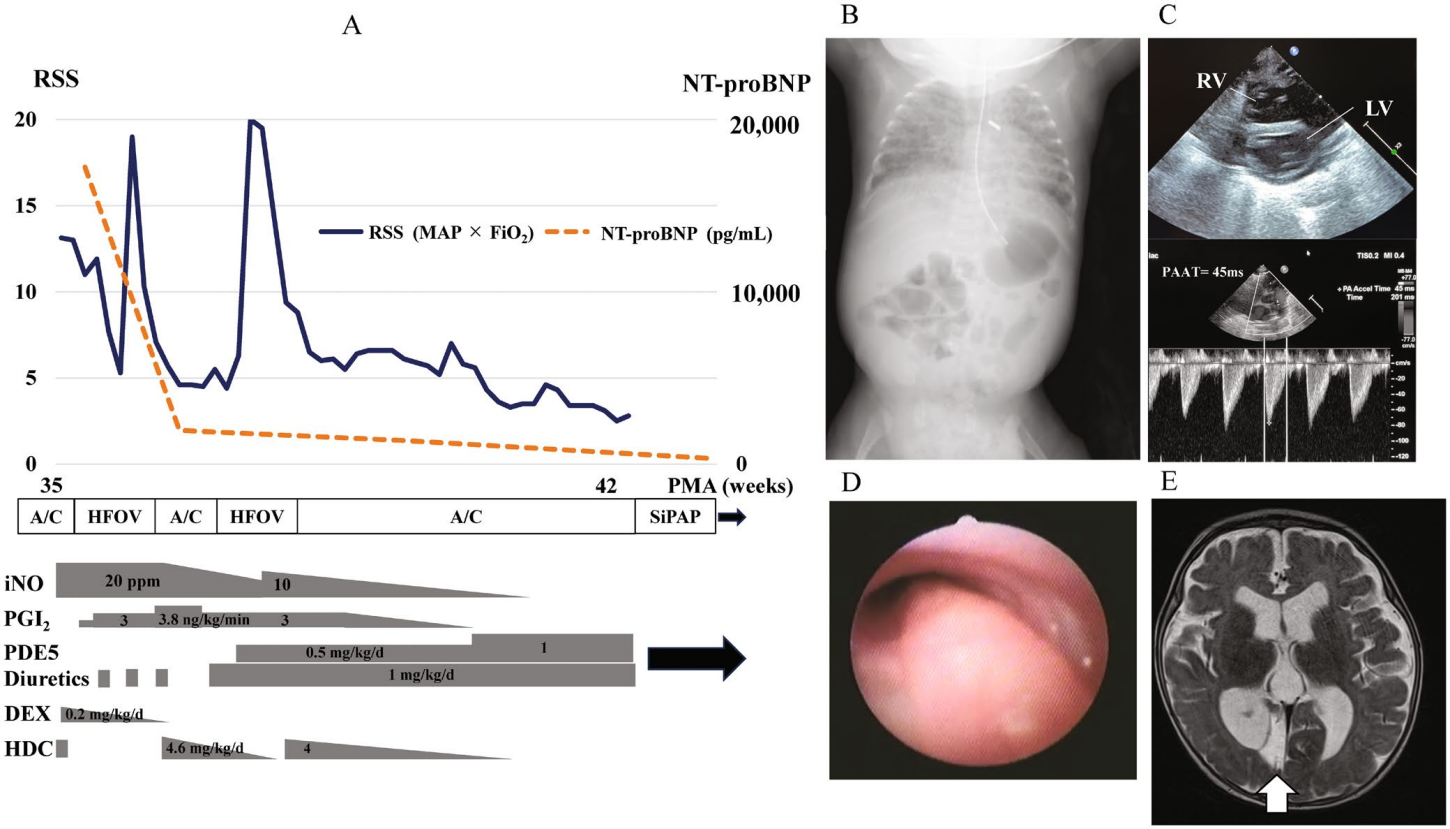
Clinical course of pulmonary hypertension treatment and associated diagnostic findings. (A) Time course of RSS and NT‐proBNP levels. (B) A radiograph of the chest and abdomen obtained at a PMA of 35 weeks. A metallic shadow consistent with surgical clipping was observed in the ductal area. (C) Echocardiography revealed interventricular septal flattening and a shortened pulmonary artery acceleration time. (D) Bronchoscopic findings at a corrected age of 3 months showing expiratory flattening of the membranous portion of the main trachea. (E) A T2‐weighted magnetic resonance image of the brain at 5 months. The arrow indicates a high‐signal lesion in the right occipital lobe. A/C, Assist/Control ventilation; DEX, dexamethasone; HDC, hydrocortisone; HFOV, high frequency oscillatory ventilation; LV, left ventricle; NT‐proBNP, N‐terminal prohormone of brain natriuretic peptide; PAAT, pulmonary artery acceleration time; PDE5, phosphodiesterase Type 5 inhibitor; PGI_2_, prostacyclin; RSS, respiratory severity score; RV, right ventricle; SiPAP, synchronized intermittent positive airway pressure.

Bronchoscopy revealed severe tracheomalacia (Figure 1D), and tracheostomy was performed at 5 months PMA. Brain MRI showed bilateral ventriculomegaly and a high‐signal lesion in the right occipital lobe (Figure 1E), indicating cerebrovascular injury.

Sildenafil (1.5 mg/kg/day) was continued with no further pulmonary complications. The lesions associated with retinopathy of prematurity remained stable, requiring no additional treatment. The infant was discharged at 8 months PMA with home ventilation and tube feeding.

The PH crisis was likely triggered by a high‐risk background of BPD‐PH and the transition from noninvasive to invasive ventilation, resulting in worsened ventilation–perfusion (V/Q) mismatch. In severe BPD, the time constant, defined as the product of lung compliance and airway resistance, is prolonged, indicating that air movement in and out of the lungs requires more time. Therefore, optimal ventilatory strategies in severe BPD typically involve a lower respiratory rate, increased tidal volume, prolonged inspiratory time, and sufficient expiratory time [4]. Although the duration of positive pressure ventilation before the onset of PH crisis in this case was relatively short, the ventilator settings might have contributed to worsening V/Q mismatch.

Ranibizumab is widely used for ROP and is considered safe and effective. However, previous reports have demonstrated that intravitreal injection of ranibizumab enters the systemic circulation and suppresses circulating VEGF levels, raising concerns regarding potential systemic adverse effects [5]. Although it remains unclear whether ranibizumab administration contributed to the PH crisis in this case, a pharmacological contribution cannot be excluded. Further accumulation of similar cases is warranted.

In summary, this case suggests that the PH crisis was likely multifactorial, reflecting severe underlying BPD, ventilatory factors, and the possible systemic effects of ranibizumab.

## Author Contributions

N.H. collected the clinical data and prepared the initial draft and figures. N.H., S.N., and T.N. participated in the clinical diagnosis and management of the patient. G.Y. critically revised the final draft. All authors reviewed and approved the final manuscript.

## Funding

The authors have nothing to report.

## Consent

Written informed consent was obtained from the patient's parents for the publication of this case report.

## Conflicts of Interest

The authors declare no conflicts of interest.

## Data Availability

Data sharing is not applicable to this article as no new data were created or analyzed in this study.

## References

[1] G. Hansmann , H. Sallmon , C. C. Roehr , et al., “Pulmonary Hypertension in Bronchopulmonary Dysplasia,” Pediatric Research 89 (2021): 446–455. 10.1038/s41390-020-0993-4 PMC7979539 32521539

[2] W. A. Blanc , “Pathology of the Placenta, Membranes, and Umbilical Cord in Bacterial, Fungal, and Viral Infections in Man,” Monographs in Pathology 22 (1981): 67–132. 7024790

[3] Y. H. Jung , J. Jang , H. S. Kim , et al., “Respiratory Severity Score as a Predictive Factor for Severe Bronchopulmonary Dysplasia or Death in Extremely Preterm Infants,” BMC Pediatrics 19 (2019): 121. 10.1186/s12887-019-1492-9 PMC6480897 31014304

[4] R. Sindelar , E. G. Shepherd , J. Ågren , et al., “Established Severe BPD: Is There a Way Out? Change of Ventilatory Paradigms,” Pediatric Research 90 (2021): 1139–1146. 10.1038/s41390-021-01558-8 34012026

[5] R. L. Avery , A. A. Castellarin , N. C. Steinle , et al., “Systemic Pharmacokinetics and Pharmacodynamics of Intravitreal Aflibercept, Bevacizumab, and Ranibizumab,” Retina 37 (2017): 1847–1858. 10.1097/IAE.0000000000001493 PMC5642319 28106709

